# The Probable Effect of Irrigation Solution and Time on Bond Strength to Coronal Dentin: An *In Vitro* Evaluation

**DOI:** 10.22037/iej.v12i4.10106

**Published:** 2017

**Authors:** Fatemeh Mokhtari, Ehsan Anvar, Mostafa Mirshahpanah, Hamidreza Hemati, Alireza Danesh Kazemi

**Affiliations:** a *Department of Endodontics, Dental School, Shahid Sadoughi University of Medical Sciences, Yazd , Iran; *; b *Private Practice, Yazd, Iran;*; c *Endodontist, Tehran, Iran;*; d * Department of Restorative Dentistry, Dental School, Shahid Sadoughi University of Medical Sciences, Yazd, Iran*

**Keywords:** Bond Strength, Dentin, Irrigation Solution

## Abstract

**Introduction:**

The aim of this study was to evaluate the effect of root canal irrigants on the microtensile bond strength of 2-step self-etch adhesive to dentin.

**Methods and Materials:**

n this study 36 sound extracted human third molars were used. After grinding 3 mm of occlusal surface, teeth were randomly divided into 6 groups based on irrigation material naming normal saline, 5.25% sodium hypochlorite (NaOCl) and 2% chlorhexidine (CHX) and also irrigation time (5 or 30 min). Next, teeth were restored with Clearfil SE bond adhesive resin system and Z250 composite. The teeth were then thermo cycled by thermo cycling machine, for 500 cycles between 5^º ^and 55^º^C with 60 sec dwell time and 12 sec transfer time. All samples were sectioned into bucco-lingual slabs. The sections were submitted to the micro tensile testing machine at a crosshead speed of 0.5 mm/min until fracture. Data was analyzed using the one-way ANOVA test with the level of significance set at 0.05.

**Results:**

Irrigation with normal saline, 5.25% NaOCl and 2% CHX for 5 or 30 min did not significantly change the microtensile bond strength of adhesive to dentin (*P*=0.729 for time and *P*=0.153 for material). However the maximum and minimum microtensile bond strength was attributed to normal saline (44.13 N) and NaOCl (31.29 N) groups, respectively.

**Conclusion:**

Iirrigation solution and time have no influence on microtensile bond strength of two-step self-etch adhesive to coronal dentin.

## Introduction

The successful root canal therapy needs a thorough cleaning, shaping and establishment of an ideal apical seal [1-4]. However root canal treated teeth could get contaminated again if the coronal restoration doesn’t provide adequate sealing and allow microleakage [5]. Thus, in addition to apical sealing, more attention must be focused on procedures performed to achieve an effective coronal sealing immediately after the completion of endodontic treatment. Therefore to limit the coronal microleakage of bonded restorative materials, the adhesive should be compatible with the structure of pulp chamber dentin. The coronal dentin commonly is in contact with different materials during endodontic treatments [6]. Root canal cleaning usually consists of a chemomechanical debridement. Elimination of pulpal tissue, organic and inorganic debris removal by using instruments and intracanal irrigants are objectives of this important step of treatment [7, 8]. However, chemical materials used in preparation of the root canals, may change the surface structure of dentine and change its adhesive properties with restorative material [9, 10]. Some SEM studies showed that irrigants mostly affect the non-mineralized phases of the dentin and cannot change the mechanical features of the mineralized dentinal components [11].

**Figure 1:**
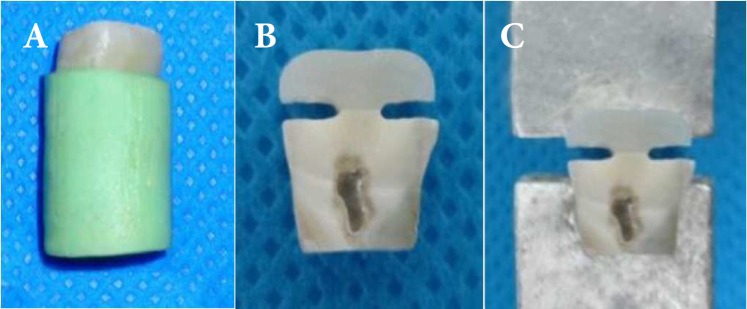
*A)* Teeth are embedded in a self-cured acrylic cylinder; *B)* Hour-glass shaped specimens; *C)* Samples are placed longitudinally in testing machine

Irrigants such as sodium hypochlorite (NaOCl), gel EDTA, chloroform and chlorhexidine (CHX) can affect the bonding strength of adhesive materials used for filling of the access cavities and when applying intracanal adhesive sealers [12, 13].

NaOCl is a widely used irrigant because it is an effective anti-microbial lubricant and has organic-tissue dissolving capabilities which facilitate root canal debridement and cleaning [11, 14]. However, there are several studies that have reported NaOCl is cytotoxic and it also could decrease the dentin-resin bond strength [15]. The main explanation is collaborated to oxidizing properties of NaOCl which can produce a rich layer of oxygen [16, 17]. This layer formed in dentin inhibits the polymerization of resin material, thus increases the microleakage [18, 19].

Another antiseptic irrigant is 2% CHX which is less cytotoxic and more substantive than NaOCl [20-22]. In several studies 2% CHX is mentioned as a material that has a broad antimicrobial effect. Also, it has numerous clinical usages such as root canal irrigation and gum disease treatment [20]. Studies showed that the action of CHX is related to its concentration [23, 24], and thus, in root canal therapy, higher concentration of CHX, such as 2% is used.

The aim of present study is to assess the effects of the root canal irrigants on the micro-tensile bond strength of adhesive material to coronal dentin.

This project was approved by the medical ethics committee of Shahid Sadoughi Dental School Research Centre. In this interventional *in vitro* study 36 extracted intact human third molars (identified under 4× magnification) were randomly selected based on previous studies and maximum facilities [24]. Teeth with caries lesions and/or discolored dentin were excluded from the study. 

Before examination teeth were cleaned off remainedtissues, disinfected in 0.5% chloramine solution (Merck, Darmstadt, Germany), and stored at 5^°^ to 10^°^C for 48 h. Then pumice powder polishing and water rinsing were done and teeth were stored in distilled water at room temperature. 

The teeth were embedded in a self-cured acrylic cylinder (Acro Pars, Tehran, Iran). Then, 3 mm of occlusal surfaces of all of the teeth were grinded by diamond cutting disc (Diamond disk, Microdont) under water cooling (dentin was not polished).

Next, the teeth were randomly divided into 6 groups of 6 teeth and immersed in different irrigants based on following protocol: Normal saline for 5 min, normal saline for 30 min (intermittent refreshing of the irrigant every 5 min), 2% CHX (Dentscare Ltda, Joinville, SC, Brazil) for 5 min, 2% CHX for 30 min (intermittent refreshing of the irrigant every 5 min), 5.25% NaOCl for 5 min, 5.25% NaOCl for 30 min (intermittent refreshing of the irrigant every 5 min).

Then all of the teeth were rinsed with distilled water for 60 sec and bonding procedure was done as described below. 

First, the self- etch primer (Clearfil SE Bond ,Kuraray, Osaka, Japan) was applied to enamel and dentin surfaces by rubbing a micro brush for 30 sec and gently air-dried for 5 sec. Next, adhesive was applied over the primer and a gentle burst of air was delivered for 5 sec. The primer was cured by light curing unit (DEMI, Kerr, Middleton, WI, USA), for 20 sec with intensity of 2500 mW/cm (the intensity of the light cure was measured by a radiometer before the examination). 

After that, Composite build-up was performed with Z250 (3M ESPE, St Paul, MN, USA) in two 1.5 mm thick increments and was cured by light cure DEMI for 40 sec with intensity of 2500 mW/cm. The teeth were then thermo cycled by thermo cycling machine (Vafaie factory ,Tehran, Iran), for 500 cycles between 5^°^ and 55^°^ C with 60 sec dwell time and 12 sec transfer time.

**Table 1:** The mean (SD) of microtensile bond strength

**Type of irrigation**	**Time**	**Mean (SD) **	**Maximum force **	**Minimum force**
**Saline**	**5 min**	38.67 (11.08)	555.16	21.97
	**30 min**	44.13 (15.40)	81.30	28.32
**5.25% NaOCl**	**5 min**	31.29 (12.81)	51.51	18.55
	**30 min**	34.22 (11.54)	64.70	22.95
**2% CHX**	**5 min**	39.70 (8.36)	55.66	28.08
	**30 min**	32.07 (11.30)	28.08	19.29

## Materials and Methods

Each tooth was buccolingually sectioned into 1 mm thickness slabs by a diamond cutting disc (Diamond disk, Microdont, São Paulo, Brazil). Then 10 slaps in each group were randomly selected. The slabs were hand-trimmed into hour-glass shaped specimens according to the technique for the microtensile bond strength evaluation.

The samples were placed longitudinally in testing machine (Micro Tester Device, Microtensile test MTD 500+/SD mechatronic GmbH, Hamm, Germany). Specimens were stressed to failure under tension at a crosshead speed of 0.5 mm per min. The required force for the fracture was recorded in MPa. In order to assess the mode of failure, the samples were evaluated by stereomicroscope (Carl Zeiss, Jena, Germany) under 40× magnification. Gathered data was analyzed by one-way ANOVA test considering the level of significance set at 0.05.

## Results

Results showed that the type of irrigant (*P*=0.153) and different irrigation times (*P*=0.729) did not have significant effect on the amount of microtensile bond strength. Also, there was no significant interaction between the two study variables (*P*=0.253). In the two-way comparison of groups, the microtensile strength was not statistically different between any of paired groups.

However, the samples immersed in normal saline for 30 min showed the highest microtensile bond strength and those immersed in 5.25% NaOCl for 5 min showed the least amount of microtensile bond strength (Table 1). 

The analysis of the fracture pattern revealed that 55 out of 60 studied samples (91.6%), had fracture line in the border of dentin and bonding. Only 3 samples (5%) showed fracture in the border of bonding and composite. Also 2 samples (3.3%) had mixed fracture pattern.

## Discussion

Endodontic irrigation solutions are commonly applied to clean and disinfect the root canal system [8]. Substances such as 5.25% NaOCl, 2% CHX and, 17% EDTA are amongst the most popular irrigants currently used in endodontics [18]. However some studies have shown that these substances can influence the quality of adhesion to dentin, therefore jeopardizing the coronal sealing and subsequently affecting the prognosis of the endodontic treatment [5, 6, 9]. This study evaluated the effect of endodontic irrigation solution and dressing procedures on the shear bond strength of composite to coronal dentin using 3 endodontic irrigants with two different irrigation times. 

Two different irrigation times (5 and 30 min) were selected because these are usually the common minimum and maximum times of the irrigant application during the root canal therapy [25]. Several studies reported that the minimum time of irrigation required to affect the intracanal bacteria such as *Enterococcus faecalis* is 3 min [26]. Therefore, setting the irrigation time 5 min or more in this study seems reasonable.

Results of this study showed that irrigation of dentin with 2% CHX, 5.25% NaOCl and normal saline either for 5 or 30 min did not significantly alter the microtensile bond strength of adhesive material to dentin.

In agreement with our findings, Santos *et al. *[6] reported that CHX had no effects on the adhesion of Clearfil SE Bond to pulp chamber dentin. Cecchin *et al.* [27] also showed that CHX pretreatment could preserve the bond strength of the fiber post relined with resin composite to root dentin. 

However, in a different study, Breschi *et al. *[21] reported that treating the dentin with CHX for 30 sec, significantly lowered the loss of bond strength and nano leakage. They also demonstrated that the CHX inhibition of dentin gelatinolytic activity (formation of MMPs) was associated with the improved bond strength of resin composite to teeth. However, the short contact time of CHX to dentin study cannot be applicable to endodontic treatments.

While the results of this study showed no remarkable effect of NaOCl on bond strength, Morris *et al.* [12] observed significantly large reduction in resin-dentin bond strength. There are other studies that obtained same results as Morris which demonstrates NaOCl could negatively affect the bond strength of adhesive material to dentin [10, 15, 28]. It is thought that NaOCl leads to oxidation of some components of dentin forming a free radical layer which inhibits the polymerization of adhesive material and subsequently lowering the bond strength [19].

Results indicated that 91.6% of specimens had fracture line in dentin-adhesive border. A possible explanation could be that the effects of irrigants on bond strength have lowered the bond strength of adhesive to dentin compared with adhesive to composite resin. Therefore in most cases fracture occurred in border of dentin to adhesive.

## Conclusion

According to the results of the present study, irrigation with 2% CHX, 5.25% NaOCl and normal saline during endodontic treatment had no adverse effects on bond strength of Clearfil SE adhesive to dentin. However further conduction of more clinical investigations with larger sample size is recommended.
